# Short‐term repeated sprint training in hypoxia improves explosive power production capacity and repeated sprint ability in Japanese international‐level male fencers: A case study

**DOI:** 10.14814/phy2.15637

**Published:** 2023-03-22

**Authors:** Masahiro Hagiwara, Takaki Yamagishi, Shogo Okamoto, Yasuyuki Azuma, Daichi Yamashita

**Affiliations:** ^1^ Department of Sport Science and Research Japan Institute of Sports Sciences, Japan High Performance Sport Center Tokyo Japan; ^2^ Albirex Niigata BB Niigata Japan

**Keywords:** explosive power production capacity, Olympic fencing, performance adaptations, repeated sprint ability

## Abstract

This case study reports the effects of six sessions of repeated sprint training in hypoxia (RSH) over 3 weeks on explosive power production capacity and repeated sprint ability (RSA) in two Japanese international‐level foil fencers. The six RSH sessions (60‐s sprints in total per session: consisting of two sets of five 6‐s sprints with 30‐s passive recovery, at simulated altitude of 3000 m) caused improvements of peak power output (PPO; Athlete A: 5.1%; Athlete B: 3.2%) and mean power output (MPO; Athlete A: 4.4%; Athlete B: 1.6%) over the 10 repeated sprints, respectively. The observed findings suggest that as few as six RSH sessions over 3 weeks can improve, at least to some extent, explosive power production capacity (PPO) and RSA (MPO) in the two elite fencers. To the best of our knowledge, this is the first study to apply short‐term RSH in combat sport (fencing) with international‐level athletes. Further studies are required to explore the effectiveness of short‐term RSH in combat sports with a more robust study design (e.g., randomized control trial with adequate statistical power) as the modality of RSH would suit physical and physiological demands in the majority of combat sports (e.g., wrestling, boxing).

## Graphical abstract

This case study reports the effects of six sessions of repeated sprint training in hypoxia (RSH) over 3 weeks on explosive power production capacity and repeated sprint ability (RSA) in two Japanese international‐level foil fencers. The six RSH sessions caused improvement of peak power output (PPO; Athlete A: 5.1%; Athlete B: 3.2%) and mean power output (MPO; Athlete A: 4.4%; Athlete B: 1.6%) over the 10 repeated sprints, respectively. The observed findings suggest that as few as six RSH sessions over 3 weeks can improve, at least to some extent, explosive power production capacity (PPO) and RSA (MPO) in the two elite fencers.

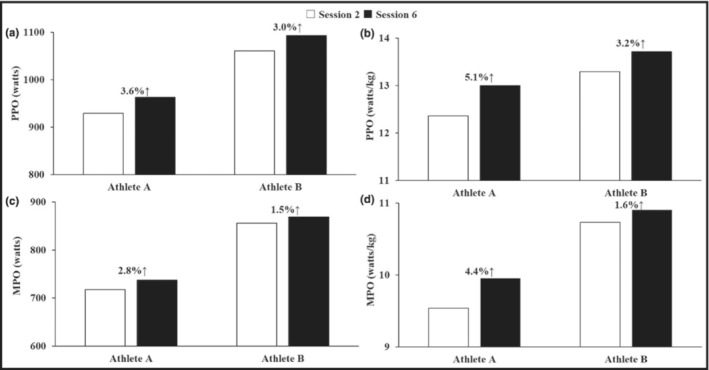

## 1 INTRODUCTION

Fencing has featured in every modern Olympic Games (OG) since 1896, and it consists of three different disciplines (i.e., épée, foil, saber). Fencing is characterized by repeated short explosive actions interspersed with low‐intensity activities (Milia et al., 2014; Roi & Bianchedi, 2008; Turner et al., 2014). Although fencing is predominantly considered as an anaerobic sport due to its repeated explosive nature (Turner et al., 2013; Turner et al., 2014), aerobic fitness should not be overlooked as enhanced aerobic capacity may favor maintaining repeated high‐intensity actions (Dupont et al., 2010). It has been shown that fencing moderately taxes aerobic and anaerobic metabolism during a simulated bout consisting of three 3‐min periods separated by 1‐min recovery in between (Milia et al., 2014). Therefore, high‐intensity interval training which taxes both aerobic and anaerobic metabolism should be desirable for success in the sport.

Recently, repeated sprint training in hypoxia (RSH) has received a great amount of attention in high‐performance sports as it likely promotes various physiological and performance adaptations in a time‐efficient manner (Beard, Ashby, Chambers, et al., 2019; Beard, Ashby, Kilgallon, et al., 2019; Brechbuhl et al., 2018, 2020; Brocherie, Girard, et al., 2015; Brocherie, Millet, et al., 2015; Faiss et al., 2015; Faiss & Rapillard, 2020). It has been reported that 2–5 weeks of RSH increased anaerobic power (Brocherie et al., 2017), maximal aerobic speed (Galvin et al., 2013), and repeated sprint ability (RSA) (Beard, Ashby, Chambers, et al., 2019; Beard, Ashby, Kilgallon, et al., 2019) to a greater extent than the same training protocol but performed in a normoxic environment in athletes of various competitive levels (e.g., elite/international, well‐trained youth). However, there is a paucity of evidence on effectiveness of RSH in preparing for major competitions such as OG in elite/international‐level athletes. Considering the abovementioned characteristics of fencing, RSH can be a useful modality to rapidly improve the ability to perform repeated high‐intensity actions during a fencing bout, potentially leading to success in the sport.

This case study reports the effects of six sessions of RSH over 3 weeks during a pre‐competitive phase of the OG season on explosive power production capacity and RSA in two Japanese international‐level male fencers.

## 2 METHODS

### 2.1 Athletes

Two elite fencers (Athlete A: height = 180.0 cm, body mass = 75.0 kg; Athlete B: height = 182.0 cm, body mass = 78.0 kg, at the pre‐training) participated in the present study. They were both in their early 20s and selected for the Japanese national foil team in the OG season; however, only Athlete A participated in the OG in the end. They can be classified as a world‐class and an elite/international‐level athletes according to their results of the OG and international competitions, respectively (McKay et al., 2022). They belonged to the Japanese national fencing team and regularly performed fencing‐specific training (approximately 3 h per session, 10 sessions per week, skill practice and sparring) and additional resistance training (approximately 2 h per day, 2 sessions per week, consisting of 14–16 exercises aiming to improve maximal strength and power for upper and lower limbs) during the study period. This study was conducted during the pre‐competitive phase in a periodized training program. This study was approved by the Institutional Research Ethics Committee (2021‐064), and a signed informed consent was obtained from both athletes.

### 2.2 Training intervention

The training intervention in this study was conducted from the middle of May to early June: the term from 68 to 49 days before the first match of the OG. Six days after the completion of training intervention, both athletes participated in a domestic competition as a final tune‐up match prior to the OG. All training sessions were conducted in the same environmentally controlled chamber with an electromagnetically braked cycle ergometer (Power Max VIII; Konami). The athletes performed repeated sprint training with maximal efforts twice per week for 3 weeks (i.e., six sessions in total) under normobaric hypoxia (F_I_O_2_: 14.5%, equivalent to a simulated altitude of 3000 m). Each athlete was exposed to nearly 30 min in hypoxic condition per training session. Thus, the total time of hypoxic exposure was approximately 180 min.

Each training session consisted of two sets of five 6‐s all‐out cycle sprints interspersed with 30‐s passive recovery (6‐min passive recovery between the sets) against the resistance of 0.075 kp per body mass. This training protocol was determined with reference to a previous study (Brocherie et al., 2017), as well as discussions among a fencing coach, a strength and conditioning coach, and two physiologists.

### 2.3 Measurement and assessment

Body mass was measured using a body scale (InBody770; InBody Japan Inc.) immediately before each training session. Before the intervention, peak power output (PPO) and mean power output (MPO) were assessed during a 30‐s all‐out sprint (Wingate test) on an electromagnetically braked cycle ergometer (Power Max VIII; Konami) against the resistance of 0.075 kp per body mass (MacDougall et al., 1998). Due to schedule constraints, this test was conducted on the day of the first training session only (i.e., no post‐intervention test). Power output during RSH was recorded throughout each training session. The PPO was defined as the highest value achieved during each training session (i.e., two sets of five 6‐s all‐out sprints), and the MPO was averaged power output over the 10 sprints. MPO was used as an indicator to assess RSA (Beard, Ashby, Chambers, et al., 2019; Beard, Ashby, Kilgallon, et al., 2019). Heart rate (HR) and arterial oxygen saturation (SpO_2_) were recorded at 1 Hz throughout the training using a wireless HR monitor (Polar H10; Polar Electro Oy) and a finger pulse oximeter (Wrist Ox_2_; Nonin Medical Inc.), respectively. The peak HR (HR_peak_) was defined as the highest HR over a 1‐s period, whereas mean HR (HR_mean_) was the value recorded during the 10 repeated sprints (i.e., excluding 6‐min passive recovery between the sets). The minimum SpO_2_ (SpO_2min_) was defined as the lowest SpO_2_ recorded over a 1‐s period and mean SpO_2_ (SpO_2mean_) was the value recorded throughout the training session.

### 2.4 Data analysis

The data of each training session were visualized graphically to identify trends in the progression and effectiveness of the six training sessions. The PPO and MPO were compared between the 2nd and 6th training sessions, and the relative changes in each value were calculated to understand the training intervention's effectiveness. The values of the first training session were not used for the comparison due to possible residual fatigue from the 30‐s Wingate test conducted prior to the session. Although we initially planned and scheduled pre‐ and post‐intervention tests, schedule constraints and limited access to our facility due to the pre‐OG training camps did not allow us to conduct post‐tests. Therefore, training effects were evaluated from the parameters obtained during the training sessions.

## 3 RESULTS

**FIGURE 1:**
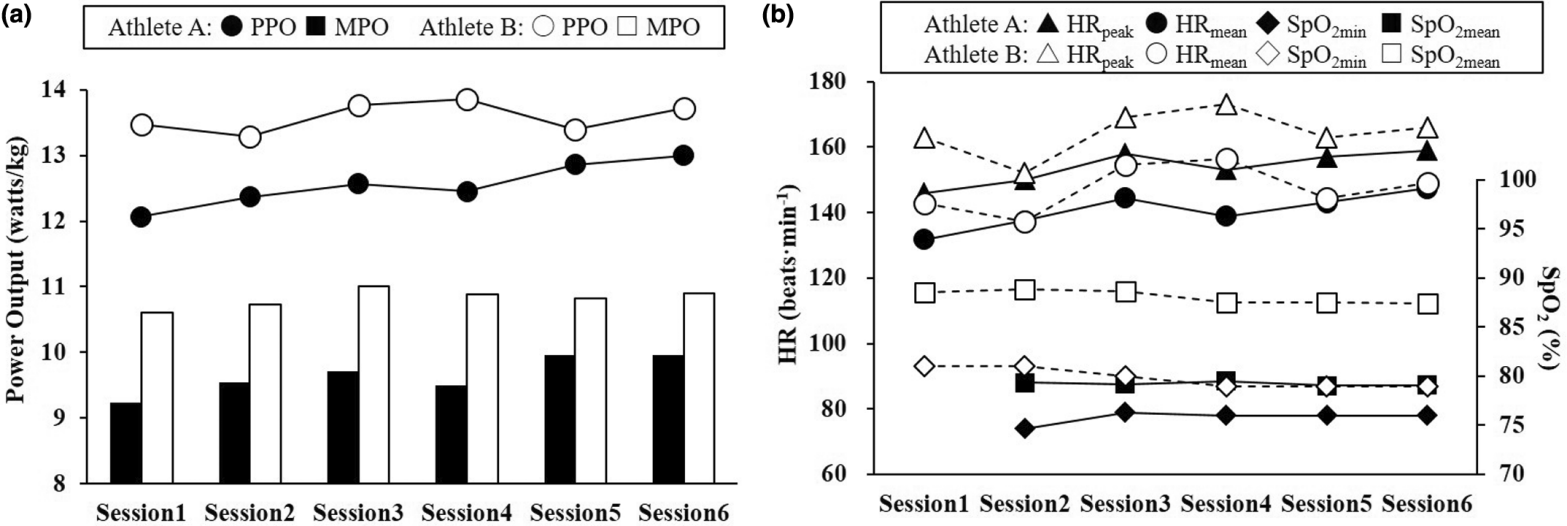
Power and physiological data during the training sessions. The best peak power output (PPO) and mean power output (MPO) during the training sessions in Athletes A and B (a). The values of heart rate (HR) and arterial oxygen saturation (SpO_2_) during the training sessions in Athletes A and B (b). HR_peak_; peak HR, HR_mean_; mean HR, SpO_2min_; minimum SpO_2_, SpO_2mean_; mean SpO_2_. *No SpO_2_ data were available due to a technical error during the first session in Athlete A.

**FIGURE 2:**
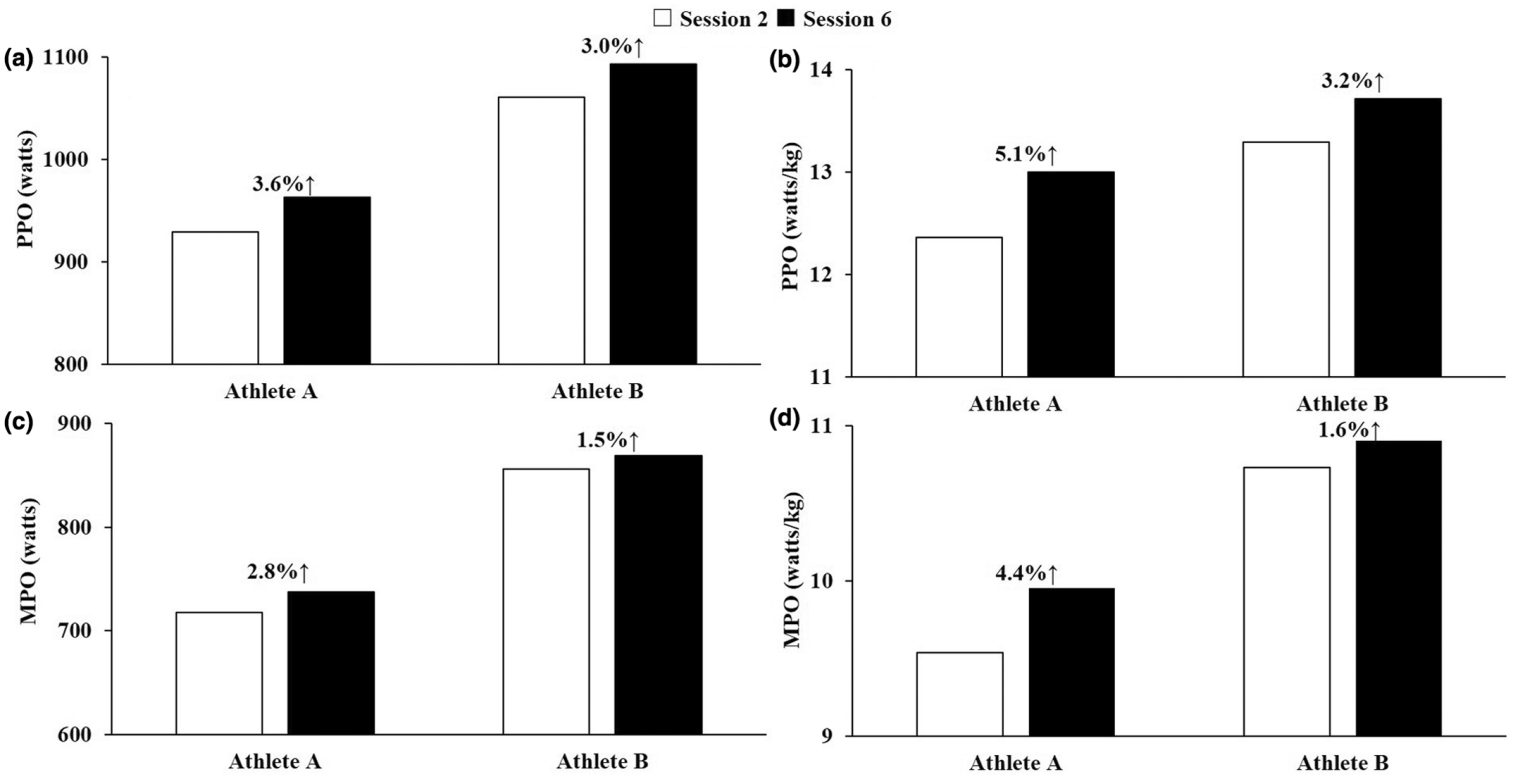
Rate of changes of peak and mean power outputs from 2nd to 6th training sessions in each athlete. The changes of peak power output (PPO) in absolute (a) and relative (b) values from 2nd to 6th sessions in each athlete. The changes of the mean power output (MPO) in absolute (c) and relative (d) values from 2nd to 6th sessions in each athlete. Numbers (%) in the graphs indicate relative change in each parameter.

The body mass of Athletes A and B at the 2nd training session was 75.2 and 79.8 kg, respectively, while that at the 6th training session was 74.1 and 79.7 kg, corresponding to changes of −1.1 kg (−1.4%) and −0.1 kg (−0.1%) for Athletes A and B, respectively. Table 1 shows the results of PPO and MPO in absolute and relative values during the 30‐s Wingate test in this study. Figure 1 shows the PPO, MPO, HR_peak_, HR_mean_, SpO_2min_, and SpO_2mean_ in all training sessions in each athlete. Both athletes completed all RSH sessions with SpO_2mean_ of below 90% and SpO_2min_ of below 80% (Figure 1b). PPO, MPO, HR_peak_, and HR_mean_ tended to gradually increase until the last session, but SpO_2min_ and SpO_2mean_ showed little change (Figure 1a,b). Figure 2 shows the rate of changes in PPO and MPO from the 2nd to 6th training sessions in each athlete. The rate of changes in absolute PPO and MPO were 3.6 and 2.8% for Athlete A, and 3.0 and 1.5% for Athlete B, respectively (Figure 2a,c). Those in relative PPO and MPO were 5.1 and 4.4% for Athlete A, and 3.2 and 1.6% for Athlete B, respectively (Figure 2b,d).

**TABLE 1:** The peak and mean power outputs during a 30‐s Wingate test. The two male (Athlete A and B) elite Japanese fencers performed a 30‐s Wingate test against the resistance of 0.075 kp per body mass.

Parameters	Peak power output (watts)	Mean power output (watts)	Peak power output (watts/kg)	Mean power output (watts/kg)
Athlete A	907	668	12.1	8.9
Athlete B	1030	796	13.0	10.0

## 4 DISCUSSION

This case study reports the effects of the training intervention consisting of six RSH sessions over 3 weeks in the pre‐competitive phase of the OG season in two Japanese elite male fencers. Both athletes tended to enhance PPO and MPO with the six RSH sessions (Figures 1 and 2) performed at a simulated altitude of 3000 m (F_I_O_2_: 14.5%), suggesting increased explosive power production capacity and RSA.

### 4.1 Indication of training effects with reference to fencing‐specific performance

The two fencers of this study were categorized as world‐class and elite/international‐level athletes (McKay et al., 2022), whose anaerobic performances achieved in the 30‐s Wingate test (Table 1) were similar compared with elite male fencers in the British team (PPO: 809 watts, 11.0 watts/kg) (Koutedakis et al., 1993) and Iranian team (MPO: 596 watts, 8.4 watts/kg) (Abdollah et al., 2014). This suggests that the RSH of this study was effective in improving explosive power production capacity (PPO) and RSA (MPO) of fencers at elite/international level who had already possessed relatively well‐developed anaerobic capacity. Fencing is characterized by repeated short explosive actions interspersed with low‐intensity activities (Milia et al., 2014; Roi & Bianchedi, 2008; Turner et al., 2014), and improving explosive power and RSA with RSH was the main purpose of this study. Although the modality of RSH in this study (i.e., cycle sprints) was not specific to that of fencing, Hamlin et al. (2017) showed that improvements of power output during RSH on a cycle ergometer ultimately corresponded to improved repeated sprint running performance. Therefore, the increased power production capacities achieved with the cycle RSH may translate into enhanced explosive and repetitive leg movements (i.e., footwork) related to offensive and defensive actions in fencing.

### 4.2 Magnitude of effects in short‐term RSH

The six RSH sessions in this study caused improvements of PPO (3.0%–5.1%) and MPO (1.5%–4.4%) over 3 weeks in the pre‐competitive phase of the OG season. It has been reported that only four sessions of RSH performed over 2 weeks during an in‐season training period increased PPO (6.3%–9.9%) and MPO (6.5%–10.8%) during repeated sprint exercises in elite/international‐level rugby players (Beard, Ashby, Chambers, et al., 2019; Beard, Ashby, Kilgallon, et al., 2019). Furthermore, Mujika et al. (2002) demonstrated that the final 3 weeks of training leading up to the OG improved swimming performance by 2.57 ± 1.45% and 1.78 ± 1.45% for male and female Olympic swimmers, respectively. Although the rates of improvements in PPO and MPO in this study tend to be lower than the previous studies (Beard, Ashby, Chambers, et al., 2019; Beard, Ashby, Kilgallon, et al., 2019), we observed comparable improvements (1.5%–5.1%) to those achieved by the Olympic swimmers in the previous study (Mujika et al., 2002). Taken together, it can be argued that the magnitude of improvement is influenced by a phase of periodization athletes are in, and even a small performance improvement (e.g., 1%–3%) would give athletes a competitive edge especially when a major competition is approaching.

### 4.3 Individual responses to RSH

This study showed that the rates of increases in PPO and MPO were slightly higher in Athlete A with lower SpO_2min_ compared to Athlete B (Figure 1b). Previous studies reported the individual variation of SpO_2_ in altitude/hypoxic training and competition (Chapman, 2013; Chapman et al., 1998). Furthermore, it has been shown that a high correlation (*r* = 0.80–0.89) exists between the reduction rate of VO_2max_ and that of SpO_2_ during maximal exercise in hypoxia (Mollard et al., 2006; Woorons et al., 2005). These findings suggest that physiological challenges (or stimuli) may be greater in an athlete with higher VO_2 max_ when performing maximal exercise in hypoxia. Although we could not obtain VO_2max_ of our athletes, the difference in SpO_2min_ between the athletes in this study (Figure 1b) may reflect difference in aerobic capacity, potentially explaining the individual variation of training effects (Chapman et al., 2014; Goods et al., 2014; Soo et al., 2020). In any case, monitoring SpO_2_ would be a useful indicator for athletes and coaches to understand an individual response to hypoxic training (Chapman, 2013).

### 4.4 Practical applications

A short‐term RSH can be performed during pre‐competitive and/or competitive phases as a way of enhancing physical and physiological capabilities toward a major competition, considering that our intervention was performed from 10 to 7 weeks prior to the OG. The RSH protocol employed in this study may be effective not only for fencers, but also for other combat athletes (e.g., wrestlers and boxers) who are required to perform repetitive high‐intensity actions with insufficient recovery.

### 4.5 Limitation

The major limitations of this study are the lack of sample size and control group, both of which did not allow us to perform a proper statistical procedure, and made it difficult to claim that we truly demonstrated the effectiveness of our intervention. Furthermore, whether increased PPO and MPO obtained from RSH actually translated into fencing performance has not been verified. Nevertheless, this study showed a possible beneficial effect of short‐term RSH in elite fencers as a way of preparation toward a major competition (e.g., OG).

## 5 CONCLUSIONS

Two elite Japanese fencers in this study tended to improve explosive power production capacity (PPO, Athlete A: 5.1%; Athlete B: 3.2%) and RSA (MPO, Athlete A: 4.4%; Athlete B: 1.6%) with six sessions of RSH at simulated altitude of 3000 m over 3 weeks. Further studies are required to explore the effectiveness of short‐term RSH in combat sports with a more robust study design.

## AUTHOR CONTRIBUTIONS

SO, TY, YA, and DY designed the study. SO, TY and YA planned training programs for the athletes. DY supervised the progress of the athletes' training. SO, TY and YA collected the data. MH, TY, SO and YA analyzed the data. MH and TY wrote the manuscript and all authors critically reviewed it. All authors read and approved the final version of the manuscript.

## FUNDING INFORMATION

The publication fee of this study was supported by Japan Institute of Sports Sciences.

## CONFLICT OF INTEREST STATEMENT

The authors declare no conflicts of interest with respect to the research, authorship, and publication of this article.

## ETHICS APPROVAL STATEMENT

This study was approved by the Institutional Research Ethics Committee (2021‐064).
